# 

**Published:** 1846-01-01

**Authors:** 


☞ The attention of the reader is invited to the remarks addressed to *Readers and Correspondents*—see cover, next page.

### THE BUFFALO MEDICAL JOURNAL

☞ Mr. T. S. CUTTING is authorized to act as Agent for this Journal, collect subscriptions, &c.

### TO READERS AND CORRESPONDENTS.

Our Journal makes its appearance on the first day of the new year. In conformity with editorial usage, and our own feelings, we tender to its patrons our cordial wishes for their health and happiness. We cannot wish them to be rendered prosperous by an increase of the prevalence of disease, but we may with propriety hope that their professional duties will be made agreeable by the kindly consideration of their patients, and that they may receive a fair proportion of the more substantial acknowledgments of the value of their labors.

Will our readers reciprocate these good wishes in behalf of the Journal? We must ourselves answer this question, and we flatter ourselves by imagining it to be in the affirmative. Acting upon this belief we beg leave to unfold somewhat of our plans for the coming year, after the present volume has expired. We commenced the publication of the Journal as an experiment to test the feasibility and propriety of the undertaking, intending it to be but preliminary to a larger work, if the views with which it was commenced should be sustained. With regard to the result of the experiment, we have only to say, that we are still more confirmed in the belief that there is a field for success and usefulness in this department of effort, and that we are encouraged to persevere, and increase our exertions in order to render the work more commensurate with the good to be effected. If a Medical Journal be desirable at this point, it should be larger, and more comprehensive in its objects. We purpose, therefore, on the first of May next, to increase its limits to nearly treble its present size, to extend its scope, and, as we trust, thereby greatly to enhance its character and usefulness. We design, also, to commence the second volume with an improved typographical appearance, so that, in this respect, it shall not be excelled by any of the medical periodicals in our country. We expect to be prepared to issue our prospectus with the next number, and will, therefore, omit at present further details, excepting to say that our plan embraces particularly analytical reviews—a province of periodical literature which we deem of especial interest and importance to the practitioner.

Our aim is to establish in this city, upon a permanent foundation, a Journal which shall be creditable to ourselves, and satisfactory to the profession. Pecuniary compensation for our labor does not, at present, at least, enter into our anticipations. Should the income exceed the expense of publication, we pledge ourselves to devote the surplus to the interests of the work. If circumstances permit, we shall offer pecuniary inducements for the contribution of sterling articles. As it is, we hope to enlist the co-operation of those who will furnish valuable contributions to its pages.

We beg leave to commend our project to the consideration of those who have signified their approbation of the present publication; and to invite those who may feel disposed to do so, to communicate with the editor with reference to the particular views which they may entertain, and to submit propositions to furnish contributions. We shall desire agencies to extend its circulation, and on this subject we shall be glad to receive applications. The success of our enterprise must depend not alone on our exertions, but on the general interest of the profession to have it succeed. As regards ourselves, all that would be proper for us to say, is, that our efforts shall not be wanting; and, on the part of our readers, if it be deemed desirable to carry out the plan contemplated, we trust they will be ready to do what they can to sustain and promote it.

We have received communications from Drs. Van Pelt and Colvin, which will appear in our next number.

The following publications are acknowledged: N. Y. Medical Intelligencer, Western Med. and Surg. Journal, British American Journal, Stockton's Dental Intelligencer, N. Y. Med. and Surg. Reporter, Boston Med. and Surg. Journal, Illinois Med. and Surg. Journal, Missouri Med. and Surg. Journal, St. Louis Med. and Surg. Journal, Bulletin of Medical Science, N. York Journal for September, Western Lancet, Journal of Insanity, Thoughts on the connection of life, mind and matter in respect to education, by J. P. Batchelder, M. D, The Chemical relations of the human body with surrounding agents, an introductory lecture by Washington L. Atlee, M. D., Prof. of Medical Chemistry in Pennsylvania College.

---

☞ The Buffalo Medical Association will hold its regular monthly meeting on Tuesday evening,—6th inst.—at the office of Dr. Flint.

A. FLINT, Recording Secretary.

